# 

**Published:** 1893-04

**Authors:** 


### CONTENTS.

ORIGINAL ARTICLES—Shock and its Treatment, by J. I. Darby, M. D., Americus, Ga. . . . . . . . 171

Removal of Cedar Pencil from Bladder of Railway Engineer, by H. L. Battle, M. D., Wadley, Ga. . . . . . . . 177

Cockleburr Removed without Instruments from Air Passage of Boy by Intra-Laryngeal Manipulations, by E. J. Sprathing, B. S., M. D., Opelika, Ala. . . . . . . . 179

Fever, by L. B. Anderson, M. D., Norfolk, Va. . . . . . . . 180

CORRESPONDENCE—New York Letter. . . . . . . . 191

SOCIETY REPORTS—Gynecological and Obstetrical Society of Baltimore 196

BOOK REVIEWS—Obstetrics, by Charles W. Hayt, M. D. . . . . . . . 205

A Practical Treatise on Diseases of the Skin, by John V. Shoemaker, A. M., M. D. . . . . . . . 205

Taylor's Medical Jurisprudence. . . . . . . . 206

SELECTIONS AND ABSTRACTS—Cystitis in Women. . . . . . . . 207

Measures for Checking the Vomiting Excited by Anæsthetics. . . . . . 208

EDITORIALS—Precautions Against Collapse from Anæsthesia. . . . . . . . 209

Remarks of Dr. J. McF. Gaston, Jr., at the Banquet of the Southern Medical Society. . . . . . . . 212

Atlanta Polyclinic. . . . . . . . 214

Meeting of the Medical Association of Georgia. . . . . . . . 215

EDITORIAL NOTES. . . . . . . . 218

BOOKS AND PAMPHLETS RECEIVED. . . . . . . . 219

PRESCRIPTIONS. . . . . . . . 220–221

SPECIAL NOTES. . . . . . . . 222–228

